# The Native Monomer of Bacillus Pumilus Ribonuclease Does Not Exist Extracellularly

**DOI:** 10.1155/2018/4837623

**Published:** 2018-10-08

**Authors:** Olga Ilinskaya, Vera Ulyanova, Irina Lisevich, Elena Dudkina, Nataliya Zakharchenko, Alexandra Kusova, Dzhigangir Faizullin, Yuriy Zuev

**Affiliations:** ^1^Department of Microbiology, Institute of Fundamental Medicine and Biology, Kazan (Volga Region) Federal University, Kazan 420008, Russia; ^2^Kazan Institute of Biochemistry and Biophysics of FRC Kazan Scientific Center of RAS, Kazan 420008, Russia; ^3^Kazan State Power Engineering University, Kazan 420066, Russia

## Abstract

Supported by crystallography studies, secreted ribonuclease of* Bacillus pumilus* (binase) has long been considered to be monomeric in form. Recent evidence obtained using native polyacrylamide gel electrophoresis and size-exclusion chromatography suggests that binase is in fact dimeric. To eliminate ambiguity and contradictions in the data we have measured conformational changes, hypochromic effect, and hydrodynamic radius of binase. The immutability of binase secondary structure upon transition from low to high protein concentration was registered, suggesting the binase dimerization immediately after translocation through the cell membrane and leading to detection of binase dimers only in the culture fluid regardless of ribonuclease concentration. Our results made it necessary to take a fresh look at the binase stability and cytotoxicity towards virus-infected or tumor cells.

## 1. Introduction

The low molecular weight guanyl-preferring ribonuclease (RNase) secreted by* Bacillus pumilus* is known due to its biological activity including antiviral and antitumor effects [1–5]. Binase, the trivial name of the enzyme, derives from the first letters of its producer strain's name,* Bacillus intermedius*, which recently has been reidentified as* B. pumilus *using molecular methods [6]. Starting from the first publications [7], binase had been considered as a monomeric cationic protein with molecular weight of 12 kDa. Today the dimerization of binase has been confirmed by direct observation of dimers in the culture fluid of the producer [8] supporting theoretical calculations of binase monomer association in solution [9]. Covalent bonds do not take part in the oligomerization of binase due to the absence of cysteine residues containing SH-groups in the molecule. Probably, the dimerization takes place during the interaction of N- or C-terminal domains (domain swapping) suggesting the presence of several dimer models which differ in stability [8].

Protein secretion in* Bacillus* is mainly mediated by the Sec system. Chaperone SecB directs the newly synthetized secreted protein towards the membrane pore SecYEG preventing its folding into a native structure [10]. The binase signal sequence of 28 amino acid residues is typical for Sec-translocated proteins and is followed by the negatively charged propeptide of 24 amino acid residues. Such small propeptides increase the effectiveness of protein secretion in* Bacillus* [11]. Proteins translocated through the SecYEG pore to the cell surface immediately acquire a native conformation to avoid proteolytic degradation. Secretion of proteins directly in a dimeric state is also possible [12, 13]. Most of them are secreted through nonclassical pathways and require an unknown three-dimensional recognition signal for secretion.

In order to understand at which stage the binase dimerization takes place, we put forward two alternative suggestions: either the enzyme is secreted by* B. pumilus* in the form of monomers and their association takes place along with an increase of protein concentration, or the preformed dimer is secreted into the extracellular environment. In favor of the first hypothesis, we can cite numerous studies on the physicochemical properties of binase performed by various research groups which actually have not mentioned the existence of aggregated forms of enzyme, as well as crystallographic studies which have not given an unambiguous answer to the question about association properties of binase [14–17]. In addition, the interaction of monomers occurs in many points of contacting surfaces supported by dozens of weak bonds (hydrophobic, ionic, and hydrogen bonds) and the enzyme accumulates gradually in the culture fluid; therefore spatial difficulties can ensure the absence of dimerization under conditions of low protein concentration.

On the other hand, the protein hydration shell can screen its polar fragments and prevent protein oligomerization. This suggests that dimers of binase found in the culture fluid [8] should be formed immediately after secretion. It should be noted that the ability to form multimers was established for the precursors of Bacillus secreted proteinases [18].

The present work was carried out to analyze the possibility of existing for natural monomeric forms of* B. pumilus *secreted RNase. Their presence will unequivocally confirm the first of our assumptions. If monomers are formed only at the stage of translation and their oligomerization occurs intracellularly or on the cell wall of Gram-positive bacilli, such data will eliminate all questions about the ambiguity and contradictions of previously obtained results concerning binase supramolecular structure.

## 2. Materials and Methods

### 2.1. Enzyme

Binase is a cationic guanyl-preferring RNase (pI 9.5) hydrolyzing RNA without a need for metal ions and cofactors. It consists of 109 amino acid residues with the molecular weight of 12.3 kDa. Binase was isolated from* B. pumilus* culture fluid and purified according to the procedure previously described for bacillary RNases [19, 20]. Catalytic activity of binase was 1.4×10^7^ U/mg when measured against high molecular weight yeast RNA at pH 8.5 and 37°C [4]. One unit of RNase activity was defined as the amount of enzyme that increases the extinction of acid-soluble products of RNA hydrolysis at 260 nm by one unit per minute measured at 37°C and pH 8.5.

### 2.2. SDS-PAGE

Protein oligomers were separated by the polyacrylamide gel electrophoresis in the presence of 0.1% SDS. Separating gel contained 15% acrylamide, and stacking gel contained 6% acrylamide. Protein samples were suspended in 4x sample buffer with final SDS concentrations of 1%, 1.5%, and 2%. Electrophoresis was performed at 150 V for 60 min. Proteins were stained with Coomassie R-250.

### 2.3. CD-Spectrometry

The Circular Dichroism (CD) spectra were measured in the 190-260 nm wavelength range on the Jasco J-1500 spectrometer (Japan) with a scanning speed of 50 nm/min. The binase was desalted and transferred into a 0.01 M sodium phosphate buffer, pH 7 using Zeba 7 kDa columns. The concentration of the stock binase solution was determined after filtration through 0.22 *µ*m pore membrane using the extinction coefficient E_280_^0.1%^ = 2.205. Quartz cuvettes with 0.01 cm and 0.1 cm optical path way were used to study enzyme concentrations 1 mg/mL and 0.05 mg/mL, respectively. The cuvettes were thermostated at 25°С. The content of the protein secondary structure elements was determined with the help of the CDSSTR algorithm (protein set 4) using DichroWeb server (http://dichroweb.cryst.bbk.ac.uk).

### 2.4. UV-Spectrometry

The absorption spectra were recorded on Lambda 25 instrument (Perkin Elmer, USA) in the wavelength range 190–400 nm at 25°C. The solutions were prepared in the same way as for CD measurements. Stock solutions in 0.01 M sodium phosphate buffer, pH 6.1 or 0.1 M K_2_SO_4_, pH 3.0 were titrated directly in a measuring 1 cm quartz cuvette with the same buffer composition. The calculations using on-line server http://protcalc.sourceforge.net/cgi-bin/protcalc showed that at these pH values binase carries the overall positive charge of +5 and +16, respectively.

### 2.5. Self-Diffusion Measurements

The NMR self-diffusion method was applied for the direct assessment of hydrodynamic size [21, 22] of binase at acidic and neutral pH. Samples were studied at protein concentration 1.5 mg/ml at pH 3.0 and pH 6.4 by dissolving the protein powder in D_2_O in order to minimize the signal from water protons in NMR spectra. ^1^H-NMR experiments were carried out on the AVANCE III NMR spectrometer (Bruker, Germany) operating at 600.13 MHz. The spectrometer was equipped with a standard z-gradient inverse probe head (TXI, 5 mm tube) capable of producing gradients with a maximum strength of 55.7 G/cm. We used a stimulated-echo sequence incorporating bipolar gradient pulses and a longitudinal eddy current delay (BPP-LED) [23]. The experimental parameters were as follows: a 90° pulse length 10-13 *μ*s; spectral width 13 ppm; time domain data points 16-32 K; the number of scans 16 recycling delay 2-5 s. The amplitude of field gradient was varied from 2% to 98% of its maximum value over 16-32 increments under constant diffusion time (Δ=50 ms) and gradient pulse duration (*δ*=6-12 ms). A gradient recovery delay of 0.1 ms and an eddy current delay of 5 ms were used. Data processing and the analysis were performed using the Bruker Topspin 3.5 software. The chemical shift region for measurement of the self-diffusion coefficient was chosen in the up-field domain of the spectrum that contained strong proton signals at 0.7-0.9 ppm.

### 2.6. Statistics

Each experiment was performed in triplicate. Statistical differences were analyzed with the standard methods using Microsoft Excel 2007.

## 3. Results

### 3.1. Electrophoresis Revealed Binase Monomers and Dimers Depending on the Protein Concentration

**Figure 1:**
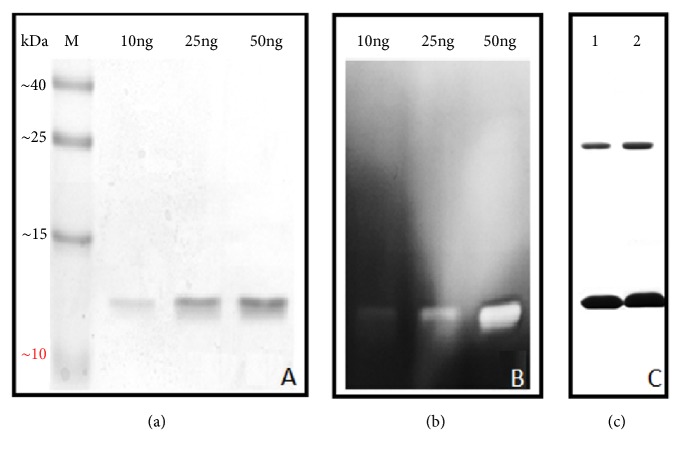
Electrophoretic study of binase. (a) Polyacrylamide gel electrophoresis (PAGE) with 0.1% SDS at nanoconcentrations of the enzyme (M, molecular weight markers). (b) Zymogram of binase at nanoconcentrations. (c) PAGE of binase at microconcentration (10 *µ*g) in the presence of strong denaturing agents: 1 - 1.5 % SDS; 2 - 2 % SDS.

Electrophoresis of binase conducted at low concentrations of enzyme (less than 50 ng) in the presence of 0.1% SDS showed no dimeric bands (Figure 1(a)). In-gel detection of catalytic activity of binase in those concentrations confirmed the presence of monomeric forms only (Figure 1(b)). However, the increase of binase concentration allowed detecting of its dimer even when SDS concentration was increased up to 2%. Moreover, the use of strong denaturing agents such as urea or guanidine chloride did not lead to disappearance of binase dimeric band indicating that micromolar enzyme concentrations facilitate the identification of binase dimers even under strong denaturing conditions (Figure 1(c)).

We can assume that the certain ratio between protein and denaturing agent allows the complete destroying of binase dimeric structure. Under the conditions when only monomers were visible, the approximate ratio SDS/protein was more than 1000 whereas upon the detection of monomers and dimers it was slightly more than 100 (calculated according to Figures 1(a) and 1(c)). It is also possible that protein forms unstable dimers at low concentrations susceptible to disintegration due to the SDS action. High binase concentrations allow the formation of highly stable dimers which do not dissociate even in the presence of denaturing agents.

Probably, the ratio of unstable binase dimers to highly stable swapping dimers which are simultaneously present in the medium is crucial. According to the data obtained for dimers of bull semen RNase (BS-RNase) the ratio of swapped to unswapped dimers is 3 : 7 [21]. The similar dependence can be assumed for binase. Therefore, at low enzyme concentrations (less than 100 ng) only monomers resulting from unswapped dimers dissociation are visible (Figures 1(a) and 1(b)). With increasing protein concentration the amount of stable swapped dimers raises making them detectable on the gel (Figure 1(c)).

### 3.2. CD Spectroscopy Indicated No Protein Conformational Transition under Increase of Binase Concentrations

**Figure 2:**
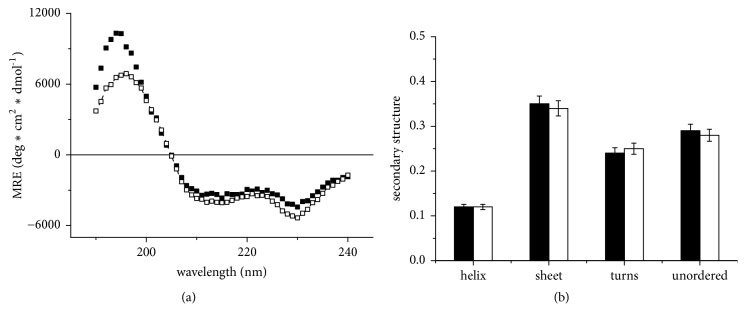
CD spectra of binase in units of molar (on a residue) ellipticity (a) and relative content of secondary structure elements of binase (b) for enzyme concentrations of 0.05 mg/ml (dark) and 1 mg/ml (light symbols), pH 7.

The comparison of binase CD spectra at two studied concentrations (0.05 mg/mL and 1 mg/mL) at pH 7 showed no significant difference in the shape of the spectra (Figure 2(a)) besides some spreading of spectral data below 200 nm. The verification measurements showed that the intensity of the 190 nm band varies depending on the buffer absorbing properties owning to decrease of signal/noise ratio due to the influence of residual salt ions which have not been fully eliminated during protein desalting. However, we did not detect the difference in the secondary structure composition for two protein concentrations (Figure 2(b)). Thus, the CD spectra indicated the absence of significant changes in the binase secondary structure and the expected monomer-dimer conformational transition at the studied concentrations.

### 3.3. Binase Did Not Exhibit a Hypochromic Effect of UV Absorption in the Concentration Range 0-50 *µ*g/mL

**Figure 3:**
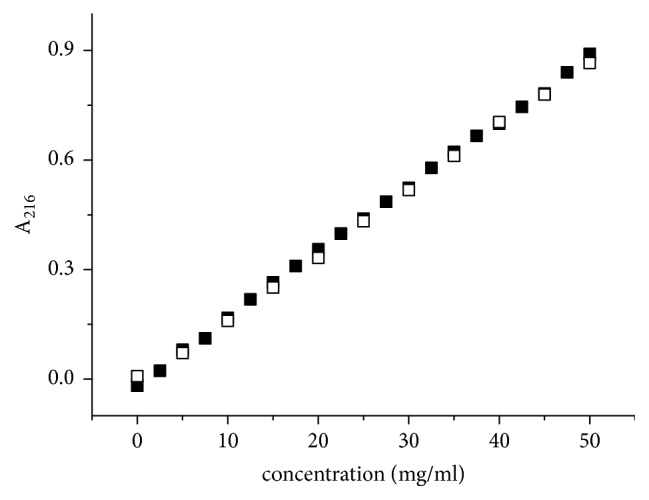
Changes in 216 nm absorbance of binase solution at pH 6.1 (light symbols) and pH 3 (dark symbols) with respect to protein concentration.

For binase a linear dependence of absorption at 220 nm on enzyme concentration in the range from 0 to 50 *µ*g/mL and pH 6.1 was established (Figure 3). Lysozyme under the similar conditions demonstrated a hypochromic effect at protein concentrations exceeding 20 *μ*g/mL [24, 25]. Based on our previous results of hydrogen exchange and NMR data [26], we assumed that the increase in the binase charge under the growth of solution acidity to pH 3.0 will shift the equilibrium between dimers and monomers and will cause the changes in solution optical properties. However, the optical density at the peptide groups absorption maximum (216 nm) had a linear dependence on concentration up to 0.05 mg/mL and the extinction was independent of the pH value (Figure 3). Thus, HE had not been observed in the studied range of binase concentration for both pH 3.0 and pH 6.1 providing an evidence for invariability of binase supramolecular structure and the lack of monomer-dimer transition.

### 3.4. Hydrodynamic Radius Supported the Existence of Binase in Dimeric Form

**Figure 4:**
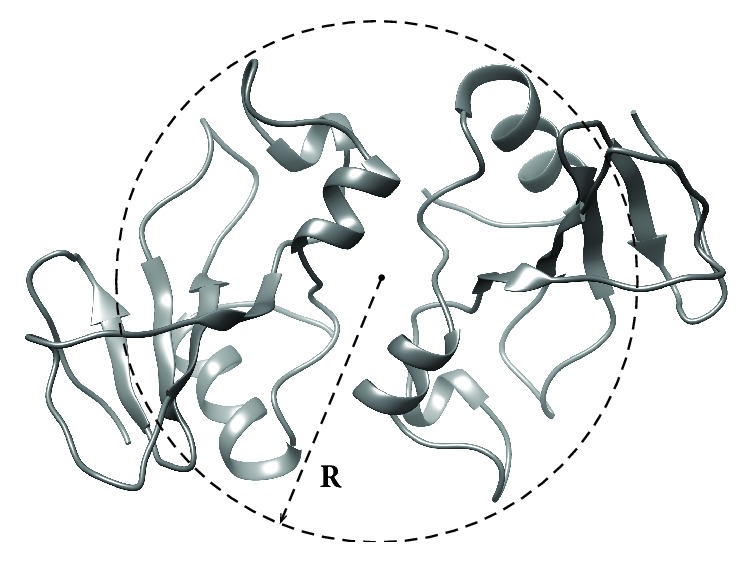
The model of binase dimer [8] and experimental hydrodynamic size (R = 2.1 nm) of protein determined by means of NMR techniques.

For water solutions the binase self-diffusion coefficients D = 1.26 × 10^-10 ^m^2^/s and D = 1.38 × 10^-10 ^m^2^/s were obtained for pH 2.8 and pH 6.4, respectively. Self-diffusion coefficient of a particle is closely related to molecular size as can be seen from the Stokes—Einstein equation:

(1) $$\begin{array}{c} D=\frac{kT}{6\pi \eta r} \end{array}$$

where* k* is the Boltzmann constant, T is the temperature, and r is particle (protein) effective hydrodynamic radius (Stokes radius) in a solution of viscosity *η*. According to this equation we obtained hydrodynamic radii of binase molecules in solutions r = 2.01 nm (pH 3.0) and r = 2.14 nm (pH 6.4).  Figure 4 depicts average hydrodynamic radius r = 2.1 nm (dashed sphere) in comparison with the results of binase computer structural modeling [8].

The theoretical size of the binase monomer globule is 1.6 nm. The increase in hydrodynamic radius by 1.2-1.3 times corresponds to the formation of the dimeric form of the protein (Figure 4) according to the relation: D = (n)^-1/3^, where D is the self-diffusion coefficient and n is the degree of oligomerization [27]. Small differences in the magnitude of the effective radii measured at pH 2.8 and pH 6.4 may be due to the differences in the extent of hydration water and the electrostatic interactions between protein molecules.

## 4. Discussion

Many RNases are prone to formation of oligomeric structures. Bovine pancreatic RNase A lyophilized from 40% acetic acid solution forms dimers, trimers, tetramers, and multimers of higher order [28, 29]. RNase of bull semen (BS-RNase), an enzyme with a molecular mass of 27 kDa and isoelectric point of 10.3, is a mixture of natural dimers of two types [30]. Dimers of the first type are formed by covalent disulfide bonds between amino acid residues Cys31 and Cys32 while prevalent dimers of the second type are additionally stabilized by exchange of N-termini [31]. Binase as well as BS-RNase, RNase A, and human pancreatic RNase H contains hydrophobic fragments capable of participating in dimerization [32], but does not have sulfur-containing amino acids for covalent cross-linking of dimers. The swapped form of BS-RNase retains its dimeric structure even if the disulfide bonds are disrupted [33]. Although the structure of RNase from* Rana pipiens* oocytes, onconase, is also stabilized during folding through disulfide bonds [34], recently discovered dimers of onconase were formed by the domain overlapping [35].

In the first works describing the oligomerization of lysozyme it was shown that the key point for this process was the ionization of amino group of active site (Glu35) which had high equilibrium constant of the acid dissociation (pKa 6.2) due to its hydrophobic environment [24]. An important characteristic of lysozyme self-aggregation was its pH dependence [36, 37]. For binase the decrease in availability of peptide groups to solvent molecules and increased intermolecular electrostatic interactions leading to partial protein association was demonstrated using the methods of hydrogen exchange and NMR relaxation with pH increasing from 2.5 to 6.0 [26]. The authors of the cited article showed that in binase crystals, where one active site was blocked, two carboxyl groups (Glu43, Glu59) were theoretically able to participate in the oligomerization. Glu43 located on the surface of protein molecule has a hydrophobic environment (Phe81, Ser37, Ser79) and high pKa value making it a potential analogue of Glu35 of lysozyme involved in the formation of dimers.

Stereospecific combination of hydrophobicity and interaction of the *β*-domains plays a key role in the oligomerization of proteins. In transition states preceding oligomerization the *α*-domain remains structured while the *β*-domain loses its secondary structure [38]. Binase, cross-linked by dimethyl suberimidate, has shown the increased hydrophobicity and lower content of *β*-structures indicating the formation of more stable dimers in comparison to the native enzyme and not the transition of monomer into dimer, as it was interpreted previously [39]. We have described two plausible models of binase dimerization, one of which is based on Van der Waals and electrostatic contacts and another is stabilized mainly by electrostatic interactions; both models provide a possibility for monomers to exchange their terminal domains [8]. As it can be seen from Figure 1, some dimeric structures were very stable and did not dissociate even in the presence of strong denaturing agents (Figure 1(c)) and the others disintegrated into monomers during SDS-electrophoresis (Figure 1(a)). It can be assumed that the presence of unstable dimers in a highly purified enzyme preparation, which were not detectable during denaturing electrophoresis of the enzyme at low concentrations, led to the fact that binase for a long time has been considered as a monomer. Our analysis of the CD spectra, the optical density at the band of peptide group absorption, and the NMR results on binase hydrodynamic radius pointed out the immutability of the protein secondary structure during the transition from low to high (up to 1.0 mg/mL) concentration and excluded the presence of a monomer in the binase preparation (Figures 2 and 3). Thus, the optical methods confirmed native dimeric structure of binase and our second assumption that* B. pumilus* secretes dimeric protein to the environment. Thanks to the Sec signal sequence binase translocates through the cell membrane in the unfolded state. Dimerization of the enzyme probably occurs during the folding or when it passes through the cell wall of* B. pumilu*s resulting in the detection of binase dimers only in the culture fluid regardless of RNase concentration [8]. Propeptides of bacterial hydrolases prevent the activation of the enzymatic function of proteins until the translocation and catalyze the folding of secreted enzymes after their transfer through the cytoplasmic membrane [40, 41]. Proteins usually pass through the cell wall by passive diffusion [10].* Bacillus* spp. use the S-layer on the surface of their cells as the three-dimensional grid for the self-assembly of secreted proteins [42]. It should be noted that the presence of such component of the cell wall as N-acetyl glucosamine does not eliminate dimerization of lysozyme [43] as it was suggested earlier [24]. This fact indirectly confirms the stability of the dimeric form of secreted binase during its diffusion through the cell wall of Gram-positive bacilli.

## 5. Conclusions

Thus, the purification of binase from the culture fluid of* B. pumilus* will always lead to the acquisition of dimers. The monomer can be obtained by the implementation of certain amino acid substitutions in the sites of binase dimerization using the method of site-directed mutagenesis. By this method a monomeric variant of natural dimer of BS-RNase with fully preserved catalytic activity and 30-fold increased cytotoxicity compared with the wild type dimer was obtained [44]. The synthetic dimer of BS-RNase constructed on its basis also demonstrated the enhanced cytotoxicity as compared with the natural dimer [44] whose antitumor activity in the reducing environment of cytosol is due to noncovalent swapped form of BS-RNase only [30, 33]. Nevertheless, the cytotoxicity of synthetic dimer was lower than that of the monomeric form. Thus, the obtaining of binase monomer opens up the possibilities for unraveling the precise mechanisms of binase antitumor action and will help to determine the deposit of the RNase supramolecular structure into its cytotoxicity.

## Data Availability

The data used to support the findings of this study are included within the article.
